# Associations of Residential Long-Term Air Pollution Exposures and Satellite-Derived Greenness with Insulin Resistance in German Adolescents

**DOI:** 10.1289/ehp.1509967

**Published:** 2016-02-05

**Authors:** Elisabeth Thiering, Iana Markevych, Irene Brüske, Elaine Fuertes, Jürgen Kratzsch, Dorothea Sugiri, Barbara Hoffmann, Andrea von Berg, Carl-Peter Bauer, Sibylle Koletzko, Dietrich Berdel, Joachim Heinrich

**Affiliations:** 1 Institute of Epidemiology I, Helmholtz Zentrum München-German Research Center for Environmental Health, Neuherberg, Germany; 2 Division of Metabolic and Nutritional Medicine, Dr. von Hauner Children’s Hospital, University of Munich Medical Center, Munich, Germany; 3 Institute of Laboratory Medicine, Clinical Chemistry and Molecular Diagnostics, University Hospital Leipzig, Leipzig, Germany; 4 IUF Leibniz Research Institute for Environmental Medicine at the University of Düsseldorf, Düsseldorf, Germany; 5 Medical School, the Heinrich Heine University of Düsseldorf, Düsseldorf, Germany; 6 Department of Pediatrics, Research Institute, Marien-Hospital Wesel, Wesel, Germany; 7 Department of Pediatrics, Technical University of Munich, Munich, Germany; 8 Division of Paediatric Gastroenterology and Hepatology, Dr. von Hauner Children’s Hospital, University of Munich Medical Center, Munich, Germany; 9 Institute and Outpatient Clinic for Occupational, Social and Environmental Medicine, University Hospital Munich, Ludwig Maximilians University Munich, Munich, Germany

## Abstract

**Background:**

Epidemiological studies have identified associations between air pollution and green space access with type 2 diabetes in adults. However, it remains unclear to what extent associations with greenness are attributable to air pollution exposure.

**Objectives:**

We aimed to investigate associations between long-term exposure to air pollution and satellite-derived greenness with insulin resistance in adolescents.

**Methods:**

A total of 837 participants of two German birth cohorts (LISAplus and GINIplus) were included in the analysis. Generalized additive models were used to determine the association of individual satellite-derived greenness defined by the Normalized Difference Vegetation Index (NDVI), long-term air pollution exposure estimated by land-use regression (LUR) models with insulin resistance (HOMA-IR) in 15-year-old adolescents. Models were adjusted for study area, cohort, socioeconomic, and individual characteristics such as body mass index, physical activity, and smoking.

**Results:**

Increases of 2 SDs in nitrogen dioxide (NO2; 8.9 μg/m3) and particulate matter ≤ 10 μm in diameter (PM10; 6.7 μg/m3) were significantly associated with 11.4% (95% CI: 4.4, 18.9) and 11.4% (95% CI: 0.4, 23.7) higher HOMA-IR. A 2-SD increase in NDVI in a 1,000-m buffer (0.2 units) was significantly associated with a lower HOMA-IR (–7.4%; 95% CI: –13.3, –1.1). Associations tended to be stronger in adolescents who spent more time outside and in those with lower socioeconomic status. In combined models including both air pollution and greenness, only NO2 remained significantly associated with HOMA-IR, whereas effect estimates for all other exposures attenuated after adjustment for NO2.

**Conclusions:**

NO2, often considered as a marker of traffic, was independently associated with insulin resistance. The observed association between higher greenness exposure and lower HOMA-IR in adolescents might thus be attributable mainly to the lower co-exposure to traffic-related air pollution.

**Citation:**

Thiering E, Markevych I, Brüske I, Fuertes E, Kratzsch J, Sugiri D, Hoffmann B, von Berg A, Bauer CP, Koletzko S, Berdel D, Heinrich J. 2016. Associations of residential long-term air pollution exposures and satellite-derived greenness with insulin resistance in German adolescents. Environ Health Perspect 124:1291–1298; http://dx.doi.org/10.1289/ehp.1509967

## Introduction

A large proportion of the global population breathes unhealthy air (van Donkelaar et al. 2010). In addition to power plants and heavy industries, urban traffic is a major source of air pollution, especially fine particulates and gaseous compounds (Krzyżanowski et al. 2005). A reduced life expectancy, mainly attributable to respiratory and cardiovascular disease, has been documented among individuals exposed to ambient air pollutants in many regions of the world (Beelen et al. 2014; Heinrich et al. 2013; Lepeule et al. 2012; Yorifuji et al. 2010). To date, no “safe limit,” at which no health effects are observed, has been identified. Indeed, exposures below the current air quality standards have been associated with adverse health effects in large cohort studies in the United States (Miller et al. 2007) and a study of > 300,000 European adults (Beelen et al. 2014).

Compared with those for all-cause mortality and cardiovascular disease, links between type 2 diabetes and air pollution have been less extensively studied in the past, even though it is believed that the same biological mechanisms—oxidative stress and systemic inflammation (Lodovici and Bigagli 2011; Thompson et al. 2010)—play a role in cardiovascular disease and type 2 diabetes development. More recently, many epidemiological studies have been summarized in systematic reviews and meta-analyses (Balti et al. 2014; Janghorbani et al. 2014; Park and Wang 2014; Thiering and Heinrich 2015; Wang et al. 2014). Taken together they provide sufficient evidence for a role of air pollution in type 2 diabetes in adults, especially for long-term exposure. We previously observed increased insulin resistance for participants with higher air pollution exposure in an analysis that included 400 children 10 years of age from the GINIplus and LISAplus birth cohorts (Thiering et al. 2013).

However, residual confounding is always possible in epidemiological studies; higher residential air pollution concentrations are often associated with lower levels of greenness. Furthermore, both factors may be associated with socioeconomic status, with effect directions depending on the study area. In addition, higher greenness (which includes green spaces such as parks and gardens, but also natural vegetation) in a neighborhood may promote a healthier lifestyle and increased physical activity while reducing other environmental impacts such as heat, noise, or ultraviolet radiation. In urban settings especially, greenness or easier access to green spaces have been linked to several diabetes-related health outcomes, such as increased physical activity (McMorris et al. 2015; Toftager et al. 2011) and well-being (Bowler et al. 2010), reduced stress (Hartig et al. 2014), improved cardiometabolic health (Paquet et al. 2013), lower blood pressure (Markevych et al. 2014), and lower body mass index (BMI) (Bell et al. 2008).

Recently, a study including > 267,000 Australian adults found a lower type 2 diabetes risk among people living in neighborhoods with more green spaces (Astell-Burt et al. 2014). However, this analysis did not confirm the prior hypothesis of the authors that better access to green spaces lowers the risk of type 2 diabetes by promoting active lifestyle and a healthier BMI. The observed reported associations for access to green space were independent of these variables. Nevertheless, it was not possible for the authors to control for air pollution exposure as well as quality and use of green spaces in their analyses. Thus, the observed association between green spaces and lower risk of type 2 diabetes may be a consequence of confounding due to lower exposure to air pollution.

In summary, for both residential greenness and air pollution, associations with metabolic diseases have been reported in adults. However, those exposures do not appear to be independent from one another. Up to now, the evidence for an association between air pollution exposure and diabetes in adults is much stronger than it is for green space, but data including both exposures are lacking. Furthermore, only a few studies exist examining the association with insulin resistance—a common precursor to diabetes—in younger ages.

Here, we aimed to investigate the effects of long-term exposure to air pollution and satellite-derived greenness on insulin resistance in 15-year-old adolescents.

## Methods

### Study Population

The study population consists of participants from two German birth cohorts in which only healthy full-term neonates with a birth weight > 2,500 g were recruited. The German Infant Study on the influence of Nutrition Intervention plus environmental and genetic influences on allergy (GINIplus) is a multi-center, two armed study consisting of 5,991 newborns recruited in maternity wards in Munich (Southern Germany) and Wesel (Western Germany). One study arm is a prospective, double-blinded, randomized intervention trial with hypoallergenic formulae, whereas the second arm is observational and does not include an intervention. The study design has been previously described in detail (von Berg et al. 2010). In the Lifestyle-related factors on the Immune System and the development of Allergies in childhood (LISAplus) study, 3,097 healthy neonates were recruited in maternity wards in Munich, Leipzig (Eastern Germany), Wesel, and Bad Honnef (Western Germany). LISAplus was designed as a population-based observational study, and children were followed up at the age of 6, 12 and 18 months and 2, 4, 6, 10, and 15 years (Zutavern et al. 2006).

Parents of all participants in LISAplus and GINIplus gave written informed consent. The studies complied with the ethical principles of the World Medical Association Declaration of Helsinki and were approved by the regional ethics committees, Bavarian Board of Physicians, and Board of Physicians of North-Rhine-Westphalia. The present analysis was covered by the original approvals and includes participants of LISAplus and GINIplus without regard to enrollment in the intervention or control arm of the original study.

The analysis is restricted to children living in the city of Munich and the adjacent regions of Upper Bavaria and Swabia (Southern Germany), and in the city of Wesel and the adjacent regions of Münster and Düsseldorf (Western Germany) (hereafter referred to as Munich and Wesel, respectively). Because no data on residential air pollution concentrations were available for the children living in Leipzig and Bad Honnef, these areas were excluded. Ultimately, the study population comprises 837 children who *a*) participated in the follow-up at age 15 years between 2011 and 2014, *b*) did not move residence between the follow-ups at ages 10 and 15 years, *c*) had information on air pollution and residential greenness, and *d*) had valid fasting measurements on insulin and glucose levels (see Figure S1). The restriction to participants who did not move in the preceding 5 years was made, because we were particularly interested in long-term effects of the exposures.

### Measurement of Insulin and Glucose

At the physical examination of the 15-year follow-up, blood was drawn after overnight fasting. Glucose measurements in blood were performed by standard laboratory methods by the two individual hospitals. Fasting insulin in serum was measured centrally by a fully mechanized system, LIAISON (DiaSorin, Saluggia, Italy). The lower limit of detection for this method was 3.5 pmol/L. Quality control samples showed intra- and interassay coefficients of variation < 5.8%. Homeostatic model assessment of insulin resistance (HOMA-IR) was calculated using the HOMA2 Calculator in Excel according to Levy et al. (1998).

### Assessment of Air Pollution Exposure

The concentrations of nitrogen dioxide (NO_2_), particulate mass of particles with an aerodynamic diameter of ≤ 10 μm (PM_10_) and ≤ 2.5 μm (PM_2.5_) and PM_2.5_ absorbance were estimated using a combination of measurements and modeling as part of the European Study of Cohorts for Air Pollution Effects (ESCAPE; http://www.escapeproject.eu/). Measurements of particulate matter were conducted at 20 monitoring sites distributed throughout each study area for three 2-week periods in cold, warm, and intermediate temperature seasons between October 2008 and July 2009. For NO_2_, parallel measurements using these 20 and additional 20 monitoring sites were performed. The annual mean concentrations of the pollutants were estimated for all residences at the time of the 15-year examination (2011–2014) using the ESCAPE area-specific land-use regression (LUR) models. A detailed description of the air pollution measurements, quality control, data analysis and the development of the LUR models has been given elsewhere (Beelen et al 2013; Cyrys et al. 2012; Eeftens et al. 2012a, 2012b).

Seven-day average concentrations before the blood draw of NO_2_, PM_10_, and PM_2.5_ were used to adjust for the potential impact of short-term air pollution exposure on insulin resistance. These average concentrations were calculated from hourly concentration data. For the Munich study area, these data were obtained from a background monitoring site located in a Munich suburban area (Johanneskirchen), which is approximately 9 km northeast of the city. For Wesel, these data were obtained from one monitoring site (WESE) that is located in the suburban area of Wesel-Feldmark, which is approximately 2 km northeast of the city.

### Assessment of Residential Greenness

Greenness was assessed using the Normalized Difference Vegetation Index (NDVI), derived from Landsat 5 Thematic Mapper (TM) satellite images (http://earthexplorer.usgs.gov/). NDVI is a common indicator of green vegetation and was developed to analyze surface reflectance measurements. Its values range from –1 to +1, with +1 indicating a high density of green leaves, –1 representing water features, and values close to zero referring to barren areas of rock, sand, or snow (Weier and Herring 2000).

Ideally, we would have assessed greenness exposure using cloud-free images for similar time periods of foliation for both study areas during 2011 corresponding to the beginning of the 15-year follow-up examinations. However, because this was not possible, we acquired cloud-free images taken during summer months with high vegetation in the year 2003 to obtain maximum exposure contrasts for both study areas. Cloud-free images for 2011 that were taken during different months for the study areas (April–May for Wesel, July for Munich) were nevertheless obtained and used in sensitivity analyses. NDVI maps were calculated based on two vegetation-informative bands [near-infrared (NIR) and visible red (RED)], available at a resolution of 30 m × 30 m, according to the formula: NDVI = (NIR – RED)/(NIR + RED). Negative pixels were excluded before further calculation. Residential greenness was defined as the mean of NDVI values in circular 500-m and 1,000-m buffers around each participant’s home address at the 15-year examination. The assignment of NDVI to the home addresses of GINIplus and LISAplus participants has been previously described (Markevych et al. 2014). We used buffer sizes of 500 m and 1,000 m because these are considered as a distance reachable within 10 and 20 min of walking and have been used in previous studies on physical activity (McMorris et al. 2015) and type 2 diabetes (Astell-Burt et al. 2014).

NDVI estimates the total vegetation. It includes green spaces such as local parks and woodland that people can enter and immerse themselves within, but also farmlands or other areas that might be green, but are not open for people to visit.

Exposure assessment was performed in ArcGIS 10.1 Geographical Information System (ESRI, Redlands, CA, USA) and Geospatial Modelling Environment (Spatial Ecology LLC) by an external company (WiGeoGIS GmbH). Geocoding was performed manually at house-accurate quality, and then the positional accuracy of the geocoded addresses was manually optimized, when necessary (< 4%). Nearly all participants of the 15-year follow-up provided addresses, and except for those residing outside of Germany, all addresses could be geocoded (98.6%).

### Covariate Assessment

All covariates were selected *a priori*. Maternal and paternal education levels were categorized based of the number of years of education (≤ 9, 10, > 10) at baseline. Equivalent net income at the 15-year follow-up was calculated by the sum of the income of all household members weighted by the size of the family. We defined city-specific income tertiles because of large differences in incomes and the cost of living between Wesel and Munich. Participants were asked how many hours per week they are physically active, lightly sweating with slightly increased respiratory rate (e.g., cycling, swimming), and how many hours they are active, heavily sweating with rapid breathing (e.g., ball sports, cardio training). Physical activity (PA), defined according to Janssen (2007), was categorized as “low” if the sum of moderate and vigorous PA performed per week was < 7 hr; as “medium” if the sum of moderate/vigorous PA was at least 7 hr; and as “high” if children performed moderate/vigorous PA at least for 10.5 hr/week and if at least 3.5 hr/week thereof were vigorous PA. In case moderate/vigorous PA was at least 10.5 hr/week, but vigorous PA was < 3.5 hr/week children were classified as “medium.” Participants were asked how many hours per day they spent outside during summer and winter, and this variable was categorized in steps of 2 hr. The pubertal development category score, derived according to Carskadon and Acebo (1993) includes three items for girls: body hair growth, breast development (not yet started = 1 point, barely started = 2 points, definitely started = 3 points, seems complete = 4 points) and menarche (yes = 4 points), who were categorized as pre-/early/mid-pubertal if the sum of the items was ≤ 4 and as late or postpubertal if the sum of the items was higher and girls already had their menarche. For boys the score includes body hair growth, voice change, and facial hair growth scored from 1 point to 4 points as mentioned above. The cutoffs for boys were pre-/early/mid-pubertal (≤ 8, but no 4 point responses) versus late or postpubertal (9–12 points).

The main analyses were adjusted for the socioeconomic covariates as separate variables. For the sensitivity subgroup analysis, we built a socioeconomic score, which was defined as the sum of paternal and maternal education levels (0 points for < 10 years, 1 point for 10 years, 2 points for > 10 years), equivalent net income (0 points for first center-specific tertile, 1 point for second center-specific tertile, 2 points for highest center-specific tertile) and secondhand tobacco smoke exposure in the home at age 15 years (0 points for every day, 1 point for sometimes, 4 points for never). For each missing value in these variables, the intermediate answer was assumed and 1 point was added to the score. This scale was subsequently categorized into tertiles and used to stratify the study population. Secondhand tobacco smoke exposure in the home at age 15 years was included in the score because it mainly represents parental smoking at home in our study, which has been shown to be correlated with socioeconomic status.

For categorical covariates except maternal and paternal education, which had a low number of missing values (0.4% and 0.8%, respectively), missing values in adjustment variables were treated as a distinct category in the analyses. In stratified analyses, participants with missing values in the variable used for stratification were excluded.

### Statistical Methods

We excluded children with HOMA-IR, NDVI, or air pollution exposures that were smaller than the mean minus four times the standard deviation (SD) or larger than the mean plus four times the SD (*n* = 6). To normalize its distribution, HOMA-IR was natural log–transformed. The Spearman correlation coefficient was used to determine the association between exposure variables. To test differences in percentages (for categorical variables) across study areas, chi-square tests were performed. To test differences in mean levels (continuous, normally distributed variables), *t*-tests were used.

Generalized additive models (GAMs), as implemented in the package “mgcv” in R (R Core Team 2013), were used to determine associations between the exposure variables and insulin resistance. These models allow the use of smooth functions for variables that may have a nonlinear relationship with the outcome (Hastie and Tibshirani 1990). The linearity of potential associations was examined using these gam models. Linearity of the association was determined by the explained deviance and estimated degrees of freedom of the smoothed terms. Because all exposure variables showed a linear association with the outcomes (see Figure S2), smoothed functions were used only for BMI and age, which showed nonlinear associations with the outcome. Basic models were adjusted for age and BMI (as smoothed functions), study area (Wesel, Munich), and cohort (LISAplus, GINIplus observation, GINIplus intervention). In addition, we evaluated models with further adjustment for smoking by the adolescent, maternal and paternal education levels, secondhand smoke in the home, physical activity, city-specific income tertiles, and pubertal development scale. Effect estimates were back-transformed from the log scale using 100 × [exp(β) – 1] and are thus presented as percent difference of the outcome, with corresponding 95% confidence intervals (CI) and *p*-values, for a 2-SD increase in the exposure variables. All tests were performed on a two-tailed significance level of 0.05.

In sensitivity analyses, we additionally adjusted the models for short term (7-day average) air pollution concentrations, performed stratified analyses, and tested interaction for the following variables: time spent outside, socioeconomic score, BMI, physical activity, and study area. Observations with missing data for the modifying variable were excluded in these analyses, and *p*-values were calculated using analysis of variance *F*-test of the model including an interaction term between modifier and exposure and the model without. In a further sensitivity analysis, we used NDVI estimated in 2011 instead of NDVI estimated in 2003. To evaluate associations between lifetime exposure and HOMA-IR, we restricted the analyses to participants who did not move between birth and the 15-year follow-up.

## Results

The characteristics of the study population combined and stratified by study area are presented in Table 1.

**Table 1:** Description of the study population.

Characteristic	All		Munich		Wesel	
	Percent or mean ± SD	*n*/*N*	Percent or mean ± SD	*n*/*N*	Percent or mean ± SD	*n*/*N*
Sex, male	51	427/837	52.2	242/464	49.6	185/373
GINIplus observation	44.6	373/837	36.6	170/464	54.4	203/373
GINIplus intervention	41.5	347/837	42.7	198/464	39.9	149/373
LISAplus	14.0	117/837	20.7	96/464	5.6	21/373
Maternal education (years)
≤ 9	9.4	79/837	8.0	37/464	11.3	42/373
10	40.7	341/837	32.1	149/464	51.5	192/373
> 10	49.5	414/837	59.5	276/464	37.0	138/373
Missing	0.4	3/837	0.4	2/464	0.3	1/373
Paternal education (years)
≤ 9	20.5	172/837	11.9	55/464	31.4	117/373
10	21.3	178/837	17.0	79/464	26.5	99/373
> 10	57.3	480/837	69.8	324/464	41.8	156/373
Missing	0.8	7/837	1.3	6/464	0.3	1/373
Equivalent net income (Euro/month)	1,727 ± 770	723	1,981 ± 799	404	1,405 ± 593	319
Smoking by the adolescent
Never	89	745/837	87.7	407/464	90.6	338/373
Sometimes	2.6	22/837	2.8	13/464	2.4	9/373
Daily	3.3	28/837	4.5	21/464	1.9	7/373
Missing	5.0	42/837	5.0	23/464	5.1	19/373
Secondhand smoke at home
Never	85.2	713/837	90.1	418/464	79.1	295/373
Sometimes	5.1	43/837	2.6	12/464	8.3	31/373
Every day	6.7	56/837	4.5	21/464	9.4	35/373
Missing	3	25/837	2.8	13/464	3.2	12/373
Socioeconomic score^*a*^
Low (0–6 points)	31.4	263/837	23.7	110/464	41.0	153/373
Medium (7–8 points)	33.2	278/837	33.0	153/464	33.5	125/373
High (9–10 points)	35.4	296/837	43.3	201/464	25.5	95/373
Age (years)	15.2 ± 0.3	837	15.3 ± 0.3	464	15.2 ± 0.3	373
BMI (kg/m^2^)	20.6 ± 3.1	837	20.4 ± 3.0	464	21.0 ± 3.2	373
Pubertal scale
Pre-/early/mid-pubertal	17.0	142/837	18.1	84/464	15.5	58/373
Late or postpubertal	64.3	538/837	65.5	304/464	62.7	234/373
Missing	18.8	157/837	16.4	76/464	21.7	81/373
Physical activity
Low	28.2	236/837	33.0	153/464	22.3	83/373
Medium	28.8	241/837	27.2	126/464	30.8	115/373
High	23.9	200/837	19.0	88/464	30.0	112/373
Missing	19.1	160/837	20.9	97/464	16.9	63/373
Time spent outside in summer (hr/day)
0–2	24.3	203/837	32.1	149/464	14.5	54/373
2.5–4	44.2	370/837	44.2	205/464	44.2	165/373
4.5–6	22.6	189/837	16.4	76/464	30.3	113/373
> 6	5.5	46/837	4.3	20/464	7.0	26/373
Missing	3.5	29/837	3.0	14/464	4.0	15/373
HOMA-IR [GM (GSD)]^*b*^	1.2 (1.6)	837	1.2 (1.6)	464	1.2 (1.6)	373
Covariates obtained at baseline: sex, study, parental education, all other covariates obtained at 15-year follow-up. ^***a***^Built from maternal and paternal education level, income, secondhand tobacco smoke in the home. ^***b***^Geometric mean (GM) and geometric standard deviation (GSD).						

Maternal and paternal education levels were higher in Munich than in Wesel (*p* < 0.001), and parents from Munich smoked less (*p* < 0.001). Because the equivalent net income was also significantly higher in Munich (*p* < 0.001), city-specific tertiles were used in the analysis with the following cutoffs: 1,548 and 2,250 EUR/month for Munich, and 1,071 and 1,500 EUR/month for Wesel. Adolescents in Wesel had higher BMIs than those in Munich (*p* = 0.012), but they also had a higher physical activity level and spent more time outside in the summer. Although the proportion of missing data was relatively high for physical activity (19.1%), pubertal scale (18.8%), and income (13.6%), it was low (0–5%) for the other covariates. No difference in HOMA-IR was observed between study centers (*p* = 0.223).

Descriptions of the exposure variables are presented in Table 2. Residential greenness was significantly higher in the more rural Wesel area than in the urbanized Munich area (all *p* < 0.001), as were all mean air pollution measures except PM_2.5_ absorbance. The latter can be explained by the fact that the Wesel study area is located near the highly industrialized Ruhr area. On the individual level, NDVI was negatively correlated with air pollutants (Table 3). All correlations between exposure variables were statistically significant except between measures of NDVI and PM_2.5_ within Munich.

**Table 2:** Mean and standard deviation of exposures.

Exposure	All		Munich		Wesel	
	Mean ± SD	*n*	Mean ± SD	*n*	Mean ± SD	*n*
NDVI 500 m buffer (NDVI units)	0.38 ± 0.09	837	0.35 ± 0.09	464	0.43 ± 0.08	373
NDVI 1,000 m buffer (NDVI units)	0.40 ± 0.09	837	0.37 ± 0.08	464	0.45 ± 0.07	373
NO_2_ (μg/m^3^)	21.3 ± 4.4	835	19.4 ± 4.7	463	23.6 ± 2.7	372
PM_10_ (μg/m^3^)	22.4 ± 3.4	837	19.9 ± 2.2	464	25.5 ± 1.3	373
PM_2.5_ (μg/m^3^)	15.1 ± 2.2	837	13.3 ± 0.8	464	17.4 ± 0.7	373
PM_2.5_ absorbance (10^–5^/m)	1.4 ± 0.2	836	1.6 ± 0.1	464	1.2 ± 0.2	372

**Table 3:** Spearman correlation of the untransformed exposures within the Munich study area (lower triangle) and Wesel study area (upper triangle).

Exposure	NDVI (500 m) (NDVI units)	NDVI (1,000 m) (NDVI units)	NO_2_ (μg/m^3^)	PM_10_ (μg/m^3^)	PM_2.5_ (μg/m^3^)	PM_2.5_ absorbance (10^–5^/m)
NDVI (500 m) (NDVI units)	1	0.86	–0.58	–0.71	–0.54	–0.6
NDVI (1,000 m) (NDVI units)	0.91	1	–0.65	–0.82	–0.64	–0.72
NO_2_ (μg/m^3^)	–0.40	–0.45	1	0.75	0.71	0.76
PM_10_ (μg/m^3^)	–0.32	–0.30	0.57	1	0.76	0.90
PM_2.5_ (μg/m^3^)	–0.15	–0.07	0.17	0.36	1	0.71
PM_2.5_ absorbance (10^–5^/m)	–0.07	–0.06	0.42	0.66	0.38	1
All correlations were statistically significant (*p* < 0.05) except between PM_2.5_ absorbance and NDVI (500 m and 1,000 m) in Munich and between PM_2.5_ and NDVI (1,000 m) in Munich.						

In the association analyses with basic adjustment (Table 4), higher greenness within a 1,000-m buffer was significantly associated with lower insulin resistance (–6.3% difference; 95% CI: –12.2, –0.1; *p* = 0.048). The estimated effect of greenness within a buffer of 500 m was similar, but did not reach statistical significance (–4.2% difference; 95% CI: –10.0, 2.0; *p* = 0.181). For the air pollution parameters, 2-SD increases of NO_2_ and PM_10_ (8.9 and 6.7 μg/m^3^, respectively) were associated with higher insulin resistance (10.6% difference; 95% CI: 3.8, 18.0; *p* = 0.002 for NO_2_, and 11.2% difference; 95% CI: 0.3, 23.3; *p* = 0.044 for PM_10_). When compared with the basic model, effect estimates tended to be stronger if socioeconomic (maternal and paternal education levels, income, secondhand smoke exposure) and individual-level factors (physical activity, time spent outside, pubertal development stage) were also considered in the analysis. However, when models were additionally adjusted for NO_2_, no environmental exposure variable except NO_2_ retained a statistically significant association with HOMA-IR. In addition, NO_2_ was still significantly associated with HOMA-IR after adjustment for NDVI (9.8% difference; 95% CI: 1.8, 18.5; *p* = 0.015 for the effect of NO_2_). Although we did not observe collinearity in the model including both NO_2_ and NDVI, some indications for moderate collinearity (increased variance inflation factors) were present in models including two air pollutants or both measures of NDVI (see Table S1). Therefore, the latter models should be cautiously interpreted. In sensitivity analyses, effect estimates were not influenced by additional adjustments for short-term air pollution concentrations in the 7 days before the day blood was drawn (see Table S2). There were no substantial differences in the effect estimates in model including the six observations identified as outliers (data not shown), using NDVI data from 2011 (data not shown), or after restricting the study population to the *n* = 414 participants who have not moved since birth (see Table S3).

**Table 4:** Associations of air pollution exposure (annual average concentrations) and NDVI (based on data from 2003) with HOMA-IR. Results of generalized additive models fitted separately for each exposure.

Exposure^*a*^	Basic model^*b*^		Further adjusted model^*c*^		Plus adjustment for NO_2_		Plus adjustment for NDVI (1,000 m)	
	% difference (95% CI)	*p-*Value	% difference (95% CI)	*p-*Value	% difference (95% CI)	*p-*Value	% difference (95% CI)	*p-*Value
NDVI (500 m)	–4.2 (–10.0, 2.0)	0.181	–5.5 (–11.3, 0.8)	0.084	–0.7 (–7.6, 6.8)	0.856	5.9 (–8.6, 22.8)	0.446
NDVI (1,000 m)	–6.3 (–12.2, –0.1)	0.048	–7.4 (–13.3, –1.1)	0.023	–2.7 (–9.9, 5.1)	0.484
NO_2_	10.6 (3.8, 18.0)	0.002	11.4 (4.4, 18.9)*	0.001			9.8 (1.8, 18.5)	0.015
PM_10_	11.2 (0.3, 23.3)	0.044	11.4 (0.4, 23.7)*	0.042	–0.4 (–13.0, 13.9)	0.948	7.0 (–4.7, 20.2)	0.251
PM_2.5_	13.2 (–3.4, 32.7)	0.128	14.6 (–2.5, 34.6)	0.099	3.9 (–13.0, 24.2)	0.672	9.1 (–7.8, 29.2)	0.309
PM_2.5_ absorbance	3.9 (–4.8, 13.4)	0.393	4.7 (–4.2, 14.3)	0.313	–5.5 (–14.9, 5.0)	0.296	1.4 (–7.7, 11.2)	0.777
^***a***^Estimates per 2-SD increase in the exposure variables: 0.2 NDVI units NDVI (500 m) and (1,000 m) 0.2 NDVI units, 8.9 μg/m^3^ NO_2_, 6.7 μg/m^3^ PM_10_, 4.4 μg/m^3^ PM_2.5_, and 0.5 10^–5^/m PM_2.5_ absorbance. ^***b***^Adjusted for: study area, cohort, sex, age, BMI. ^***c***^Adjusted for: study area, cohort, sex, age, BMI, smoking by the adolescent, maternal and paternal education levels, secondhand smoke at home, physical activity, income, pubertal scale.								

**Figure 1:**
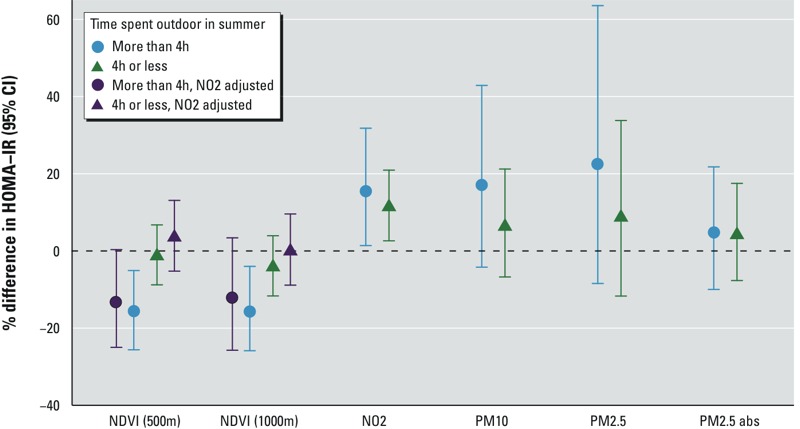
Stronger effect estimates in adolescents spending more time outside in summer. GAMs were adjusted for study area, cohort, sex, age, BMI, smoking by the adolescent, paternal and maternal education levels, secondhand smoke in the home, physical activity, pubertal state, city-specific equivalent net income tertiles. *p*-Values for the interaction with time spent outside in summer: NDVI (500 m): *p* = 0.043, NDVI (1,000 m): *p* = 0.124, NO_2_: *p* = 0.504, PM_10_: *p* = 0.885, PM_2.5_: *p* = 0.784, PM_2.5_ absorbance: *p* = 0.306.

**Figure 2:**
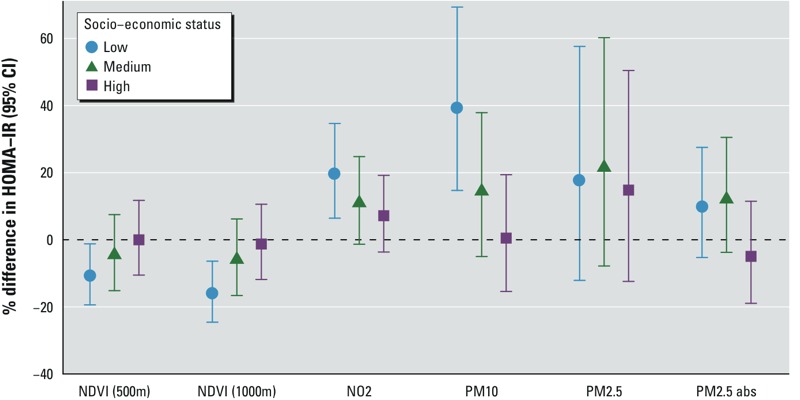
Stronger effect estimates in adolescents with lower socioeconomic score built from paternal and maternal education levels, equivalent net income and secondhand tobacco smoke exposure in the home. Gam models adjusted for study area, cohort, sex, age, BMI, smoking by the adolescent, physical activity, pubertal state. *p*-Values for the interaction with time spent outside in summer: NDVI (500 m): *p* = 0.317, NDVI (1,000 m): *p* = 0.251, NO_2_: *p* = 0.122, PM_10_: *p* = 0.029, PM_2.5_: *p* = 0.186, PM_2.5_ absorbance (abs): *p* = 0.126.

When the analysis was stratified by the amount of time the children spent outside in the summer, we observed a tendency for stronger effect estimates for all exposure variables among children who spent > 4 hr/day outside compared with children who spent < 4 hr/day outside (Figure 1). A formal statistical test of the interaction was significant for NDVI (500 m), *p* = 0.043. In addition, effect estimates for NDVI were only marginally attenuated by additional adjustment for NO_2_ in participants who spent > 4 hr/day outside. Furthermore, we observed a tendency for higher negative associations for NDVI, as well as higher positive associations for NO_2_ and PM_10_, among children with a lower socioeconomic status (Figure 2), but the interaction term was not statistically significant. Effect estimates were similar across study areas, and no significant interaction was observed between the exposure variables and study area (see Figure S3). We also did not observe a strong indication for effect modification of physical activity or BMI (see Figures S4 and S5).

## Discussion

We investigated associations of long-term air pollution exposure and satellite-derived greenness on insulin resistance in 837 adolescents 15 years of age and identified significant associations between NO_2_, PM_10_, and greenness within a 1,000-m buffer with insulin resistance. These associations were present after adjustment for other factors, such as physical activity, BMI, socioeconomic status, and secondhand tobacco smoke exposure. However, only the association between NO_2_ and insulin resistance remained significant after mutual adjustment for residential greenness and other air pollutants. Our results thus suggest that the association between insulin resistance and greenness was at least partly attributable to confounding by exposure to air pollution.

Our finding of a positive association between air pollution concentrations and insulin resistance are in line with several other studies that reported associations between air pollution and type 2 diabetes prevalence and incidence in adults (Chen et al. 2013; Coogan et al. 2012; Eze et al. 2014; Krämer et al. 2010). In a previous analysis within the GINIplus and LISAplus cohorts, we also reported a positive association between insulin resistance at age 10 years and air pollution concentrations assessed to the birth address (Thiering et al. 2013). Given that the overlap in participants between this past study and the current one is small (13.9%, 117/837), the present results provide new additional support for an association between air pollution and insulin resistance in adolescents. A study comprising 374 children living in Isfahan, Iran, who were 10–18 years of age reported associations of short-term PM_10_ concentration (within the previous 7-days) with insulin resistance (Kelishadi et al. 2009), and an experimental study reported increased insulin resistance among 25 healthy adults living in rural Michigan after they were brought to an urban location for 5 consecutive days (Brook et al. 2013). Because these studies demonstrated associations between temporal changes in the air pollution concentrations and insulin resistance, we conducted a sensitivity analysis including short-term air pollution measures in addition to long-term exposure.

To our knowledge, no previous study has reported on potential associations between greenness and insulin resistance in adolescents. A single study comprising > 267,000 adults in Australia observed a lower type 2 diabetes risk for people living in neighborhoods with more green spaces (Astell-Burt et al. 2014). Consistent with this finding, we observed a significant association between NDVI and insulin resistance at age 15 years in our analyses. However, in contrast to the Australian study by Astell-Burt et al. (2014), we were able to account for air pollution exposures in the models and found that associations with residential greenness were explained by air pollution exposure.

Although several biological mechanisms have been proposed to explain the adverse effects of air pollution exposure on the human body, such as systemic inflammatory responses and increased oxidative stress (Lodovici and Bigagli 2011; Thompson et al. 2010), impaired endothelial function, changes in autonomic nervous system functions (Genc et al. 2012), and epigenetic changes (Bind et al. 2012), the mechanisms underlying the potential health effects of residential greenness are less clear.

One hypothesized mechanism is related to increased physical activity and a healthier lifestyle among people living in greener neighborhoods. Previous studies have reported that green spaces and greenness are associated with increased physical activity: Participants living closer to green spaces had higher odds of (self-reported) use of green spaces to exercise in the Danish National Health Interview Survey 2005 (Toftager et al. 2011). In the 2001 Canadian Community Health Survey, participants in the highest quartile of greenness measured by NDVI were more likely to participate in leisure-time physical activity (McMorris et al. 2015). This trend was also observed in a study comprising 8- to 14-year-old children living in California, in which NDVI was positively associated with objectively measured physical activity (GPS and accelerometry), after excluding physical activity at home and during school hours (Almanza et al. 2012). In our analyses, we adjusted for BMI and overall physical activity and also tested for interactions with these factors, but found no indication for effect modification. Although no information about the actual use of green spaces for exercise was available in our study, we do not believe that the association between residential greenness and insulin resistance in our data is strongly confounded by increased physical activity or lower BMI in children living in greener environments because we adjusted for both factors and the association persisted. This is in line with the results of Astell-Burt et al. (2014), in which the association between green spaces and type 2 diabetes in adults was independent of physical activity.

Another hypothesis to explain potential associations between greenness and improved health is that greener areas have lower levels of harmful environmental exposures, such as heat, noise, or air pollutants (Hartig et al. 2014). For example, noise has also been linked to type 2 diabetes (Sørensen et al. 2013) even after adjustment for air pollution exposure. In our data, we observed a negative correlation between residential greenness and air pollution concentrations. Furthermore, when we additionally adjusted the analyses for NO_2_, the association between residential greenness and insulin resistance was attenuated. This result could indicate that increased air pollution concentrations in neighborhoods with little greenness could be one of the causal factors driving the observed associations with greenness in our data.

### Strength and Limitations

Major strengths of our study include the combined assessment of both air pollution exposure and residential greenness on HOMA-IR, and the available data on multiple socioeconomic and personal lifestyle factors, which allowed us to cautiously adjust the models for possible confounders. Furthermore, information on secondhand smoke exposure in the home was also available, and represents a major source of indoor air pollution. Because our study was conducted in adolescents, results might be less confounded by other factors such as preexisting illness, exposures at the workplace, and alcohol consumption. However, the possibility that residual confounding may be affecting the results can never be completely eliminated in an observational study. We observed stronger negative associations between NDVI and insulin resistance among adolescents who spent more time outside during the summer, which strengthens our belief that the observed associations are not solely attributable to residual confounding. A further strength is related to the individual-level estimation of air pollution concentrations and residential greenness at the home addresses, which was performed using state-of-the-art methodologies.

However, this study is not without limitations. Our study population is not a representative sample of German adolescents in this age group due to several reasons: *a*) loss for follow-up in the birth cohorts, *b*) potential differences in the moving behavior, and *c*) compliance with the voluntary clinical examination and blood draw. Overall, adolescents of families with a lower level of education and income were underrepresented in this analysis. However, as we observed higher effect estimates in this group we assume that our results for the total population may be more likely biased toward the null than overestimated. There is also a potential for exposure misclassification, because the modeling of air pollution concentrations at the 15-year home addresses was based on air pollution measurements that were performed about 5 years earlier. Therefore, changes in infrastructure, such as bypasses or additional housing blocks, as well as changes in pollution sources might have interfered with the estimation of the air pollution concentrations at the residential addresses. However, several studies have demonstrated that the spatial contrast of air pollution remains stable over 10 years (Eeftens et al. 2011; Wang et al. 2013). A further limitation is that data on the quality of green spaces were not available. Furthermore, data on road traffic noise were available only for a small subgroup living in the city of Munich, which made it impossible to include this information. For the satellite-derived variables, it was not possible to obtain the required cloud-free images during maximum foliation for both study areas at the time of the 15-year follow-up. However, we assume that the spatial contrasts of greenness remained stable, as our findings were replicated when alternative cloud-free days in 2011 were used, and the correlation between NDVI estimated in 2011 and NDVI estimated in 2003 was high (between 0.796 and 0.850). To reduce potential exposure misclassification, we included in the analysis only adolescents who did not move since the follow-up at age 10 years. Still, the time spent at the home address or nearby may considerably vary between adolescents.

## Conclusions

Although the associations with other exposures were attenuated after mutual adjustment, NO_2_—often considered a marker of traffic—was independently associated with insulin resistance. The observed association between higher greenness exposure and lower HOMA-IR in adolescents might thus be attributable mainly to the lower co-exposure to traffic-related air pollution.

## Supplemental Material

(3.3 MB) PDF
