# Supplementary material for: Associations of Residential Long-Term Air Pollution Exposures and Satellite-Derived Greenness with Insulin Resistance in German Adolescents
Source: Environ Health Perspect. 2016 Feb 5;124(8):1291–8. doi: 10.1289/ehp.1509967 (PMC4977044; doi:10.1289/ehp.1509967)
Supplement: (3.3 MB) PDF [file ehp.1509967.s001.acco.pdf]

Environ Health Perspect
DOI: 10.1289/ehp.1509967

Note to readers with disabilities: *EHP* strives to ensure that all journal content is accessible to all readers. However, some figures and Supplemental Material published in *EHP* articles may not conform to 508 standards due to the complexity of the information being presented. If you need assistance accessing journal content, please contact ehp508@niehs.nih.gov. Our staff will work with you to assess and meet your accessibility needs within 3 working days.

Supplemental Material

Associations of Residential Long-Term Air Pollution Exposures and Satellite-Derived Greenness with Insulin Resistance in German Adolescents

Elisabeth Thiering, Iana Markevych, Irene Brüske, Elaine Fuertes, Jürgen Kratzsch, Dorothea Sugiri, Barbara Hoffmann, Andrea von Berg, Carl-Peter Bauer, Sibylle Koletzko, Dietrich Berdel, and Joachim Heinrich

Table of Contents

Table S1. Associations of air pollution exposure (annual average concentrations) and NDVI (based on data from 2003) with HOMA-IR including additional adjustment for other exposure variables. Results of generalized additive models fitted separately for each exposure.

Table S2. Associations of air pollution exposure (annual average concentrations) and NDVI (based on data from 2003) with HOMA-IR including additional adjustment for short term (7-day average) air pollution concentrations. Results of generalized additive models fitted separately for each exposure.

Table S3. Associations of air pollution exposure (annual average concentrations) and NDVI (based on data from 2003) with HOMA-IR restricted to participants who have not moved since birth (n=414). Results of generalized additive models fitted separately for each exposure.

Figure S1. Flow chart of the study population

Figure S2. Smoother plots showing the linear association between air pollutants (annual average concentrations) and NDVI (based on data from 2003) and insulin resistance. Gam models adjusted for city, cohort, sex, age, BMI.

Figure S3. Comparable effect estimates in both cities. Gam models adjusted for cohort, sex, age, BMI, smoking by the adolescent, paternal and maternal education level, secondhand smoke in the home, physical activity, pubertal state, city-specific equivalent net income tertiles.

Figure S4. Gam models stratified for physical activity level adjusted for cohort, city, sex, age, BMI, smoking by the adolescent, paternal and maternal education level, secondhand smoke in the home, pubertal state, city-specific equivalent net income tertiles.

Figure S5. Gam models stratified for sex-specific BMI tertiles adjusted for cohort, city, sex, age, smoking by the adolescent, paternal and maternal education level, secondhand smoke in the home, pubertal state, physical activity, city-specific equivalent net income tertiles.

Table S1. Associations of air pollution exposure (annual average concentrations) and NDVI (based on data from 2003) with HOMA-IR including additional adjustment for other exposure variables. Results of generalized additive models fitted separately for each exposure.

Exposure[a]	% difference (95% CI)	p
**Further adjusted model[b]**		
NDVI (500m)	-5.5 (-11.3, 0.8)	0.084
NDVI (1000m)	**-7.4 (-13.3, -1.1)**	**0.023**
NO_2	**11.4 (4.4, 18.9)**	**0.001**
PM_{10}	**11.4 (0.4, 23.7)**	**0.042**
$PM_{2.5}$	14.6 (-2.5, 34.6)	0.099
$PM_{2.5}$ abs.	4.7 (-4.2, 14.3)	0.313
**Plus adjustment for NDVI (500m)**		
NDVI (500m)		
NDVI (1000m)	-12.2 (-24.6, 2.3)	0.094
NO_2	**10.8 (3.0, 19.3)**	**0.007**
PM_{10}	8.8 (-3.0, 22.1)	0.151
$PM_{2.5}$	11.0 (-6.2, 31.3)	0.226
$PM_{2.5}$ abs.	2.5 (-6.5, 12.3)	0.603
**Plus adjustment for PM_{10}**		
NDVI (500m)	-3.5 (-10.0, 3.5)	0.319
NDVI (1000m)	-5.8 (-12.4, 1.4)	0.112
NO_2	**11.3 (2.5, 20.8)**	**0.011**
PM_{10}		
$PM_{2.5}$	6.9 (-11.6, 29.3)	0.491
$PM_{2.5}$ abs.	-3.0 (-13.7, 9.0)	0.610
**Plus adjustment for $PM_{2.5}$**		
NDVI (500m)	-4.4 (-10.6, 2.1)	0.181
NDVI (1000m)	-6.5 (-12.7, 0.1)	0.056
NO_2	**10.5 (3.0, 18.5)**	**0.005**
PM_{10}	9.0 (-3.6, 23.4)	0.171
$PM_{2.5}$		
$PM_{2.5}$ abs.	-0.1 (-10.3, 11.3)	0.986
**Plus adjustment for $PM_{2.5}$ abs.**		
NDVI (500m)	-5.2 (-11.3, 1.3)	0.114
NDVI (1000m)		
NO_2	**-7.3 (-13.5, -0.6)**	**0.032**
PM_{10}	**13.8 (5.4, 22.9)**	**0.001**
$PM_{2.5}$	14.7 (-0.3, 31.9)	0.054
$PM_{2.5}$ abs.	16.2 (-4.9, 41.9)	0.142

[a] estimates per 2SD increase in the exposure variables: 0.2 NDVI units NDVI (500m) and (1000m) 0.2 NDVI units, 8.9 µg/m^3 NO_2, 6.7 µg/m^3 PM_{10}, 4.4 µg/m^3 PM_{25}, and 0.5 10^{-5}/m $PM_{2.5}$ abs.

[b] adjusted for: study area, cohort, sex, age, BMI, smoking by the adolescent, maternal and paternal education levels, second hand smoke at home, physical activity, income, pubertal scale

Table S2. Associations of air pollution exposure (annual average concentrations) and NDVI (based on data from 2003) with HOMA-IR including additional adjustment for short term (7-day average) air pollution concentrations. Results of generalized additive models fitted separately for each exposure.

Exposure[a]	% difference (95% CI)	p
**Further adjusted model[b]**		
NDVI (500m)	-5.5 (-11.3, 0.8)	0.084
NDVI (1000m)	**-7.4 (-13.3, -1.1)**	**0.023**
NO_2	**11.4 (4.4, 18.9)**	**0.001**
PM_{10}	**11.4 (0.4, 23.7)**	**0.042**
$PM_{2.5}$	14.6 (-2.5, 34.6)	0.099
$PM_{2.5}$ abs.	4.7 (-4.2, 14.3)	0.313
**Plus adjustment for 7-day average NO_2**		
NDVI (500m)	-5.7 (-11.6, 0.5)	0.071
NDVI (1000m)	**-7.6 (-13.6, -1.3)**	**0.0198**
NO_2	**11.7 (4.7, 19.3)**	**0.0009**
PM_{10}	**12.1 (0.9, 24.5)**	**0.033**
$PM_{2.5}$	15.5 (-1.8, 35.8)	0.081
$PM_{2.5}$ abs.	5.5 (-3.6, 15.4)	0.243
**Plus adjustment for 7-day average PM_{10}**		
NDVI (500m)	-5.4 (-11.3, 0.8)	0.085
NDVI (1000m)	**-7.4 (-13.3, -1.1)**	**0.023**
NO_2	**11.2 (4.2, 18.6)**	**0.0013**
PM_{10}	**11.7 (0.7, 24.0)**	**0.036**
$PM_{2.5}$	15.4 (-1.7, 35.5)	0.080
$PM_{2.5}$ abs.	6.3 (-2.9, 16.3)	0.186
**Plus adjustment for 7-day average $PM_{2.5}$**		
NDVI (500m)	-5.4 (-11.2, 0.8)	0.087
NDVI (1000m)	**-7.4 (-13.3, -1.0)**	**0.024**
NO_2	**11.3 (4.3, 18.7)**	**0.0012**
PM_{10}	**11.7 (0.7, 24.0)**	**0.036**
$PM_{2.5}$	15.5 (-1.6, 35.6)	0.079
$PM_{2.5}$ abs.	6.3 (-2.9, 16.3)	0.185

[a] estimates per 2SD increase in the exposure variables: 0.2 NDVI units NDVI (500m) and (1000m) 0.2 NDVI units, 8.9 µg/m^3 NO_2, 6.7 µg/m^3 PM_{10}, 4.4 µg/m^3 $PM_{2.5}$, and 0.5 10^{-5}/m $PM_{2.5}$ abs.

[b] adjusted for: study area, cohort, sex, age, BMI, smoking by the adolescent, maternal and paternal education levels, second hand smoke at home, physical activity, income, pubertal scale

Table S3. Associations of air pollution exposure (annual average concentrations) and NDVI (based on data from 2003) with HOMA-IR restricted to participants who have not moved since birth (n=414). Results of generalized additive models fitted separately for each exposure.

**Exposure**[a]	**% difference (95% CI)**	**p**
**Basic model**[b]		
NDVI (500m)	-3.1 (-11.4, 6.0)	0.492
NDVI (1000m)	-5.8 (-14.3, 3.5)	0.217
NO_2	**10.6 (0.9, 21.4)**	**0.033**
PM_{10}	12.2 (-4.6, 31.9)	0.166
$PM_{2.5}$	14.2 (-10.1, 45.1)	0.275
$PM_{2.5}$ abs.	8.1 (-5.4, 23.6)	0.252
**Further adjusted model**[c]		
NDVI (500m)	-4.0 (-12.3, 5.2)	0.385
NDVI (1000m)	-6.5 (-15.1, 3.0)	0.176
NO_2	**12.2 (2.1, 23.3)**	**0.017**
PM_{10}	13.2 (-4.1, 33.6)	0.143
$PM_{2.5}$	17.6 (-7.8, 49.9)	0.192
$PM_{2.5}$ abs.	10.2 (-3.8, 26.2)	0.161
**Plus adjustment for NO_2**		
NDVI (500m)	1.8 (-8.2, 12.9)	0.730
NDVI (1000m)	-1.4 (-11.8, 10.3)	0.804
NO_2		
PM_{10}	1.2 (-18.3, 25.3)	0.913
$PM_{2.5}$	8.6 (-17.3, 42.6)	0.556
$PM_{2.5}$ abs.	0.5 (-15.1, 19.0)	0.953
**Plus adjustment for NDVI (1000m)**		
NDVI (500m)	10.0 (-10.3, 34.9)	0.360
NDVI (1000m)	-6.5 (-15.1, 3.0)	0.176
NO_2	11.4 (-0.1, 24.2)	0.052
PM_{10}	9.0 (-10.1, 32.2)	0.379
$PM_{2.5}$	12.1 (-13.6, 45.5)	0.392
$PM_{2.5}$ abs.	7.0 (-8.0, 24.6)	0.381

[a] estimates per 2SD increase in the exposure variables: 0.2 NDVI units NDVI (500m) and (1000m) 0.2 NDVI units, 8.9 µg/m^3 NO_2, 6.7 µg/m^3 PM_{10}, 4.4 µg/m^3 $PM_{2.5}$, and 0.5 10-5/m $PM_{2.5}$ abs.

[b] adjusted for: study area, cohort, sex, age, BMI

[c] adjusted for: study area, cohort, sex, age, BMI, smoking by the adolescent, maternal and paternal education levels, second hand smoke at home, physical activity, income, pubertal scale

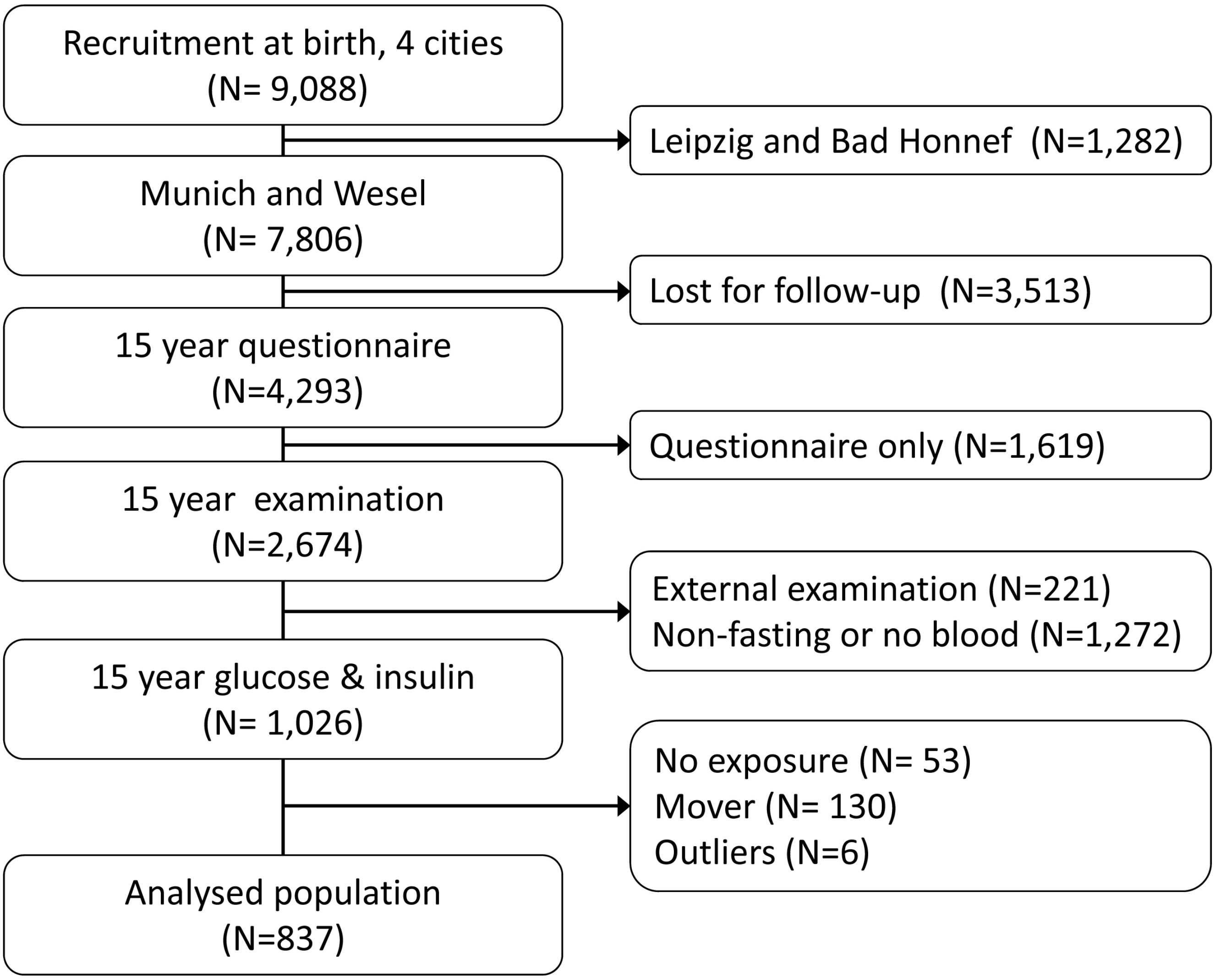

Figure S1. Flow chart of the study population

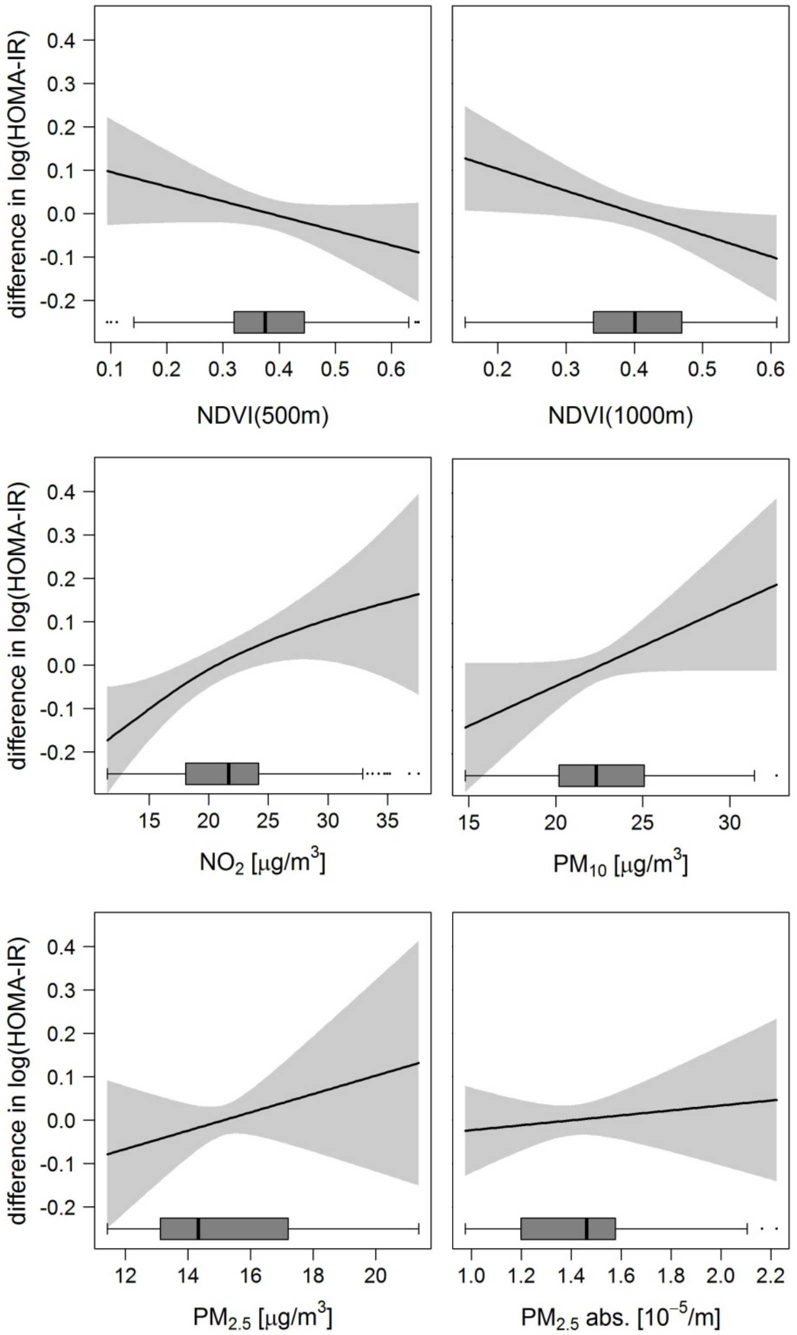

Figure S2. Smoother plots showing the linear association between air pollutants (annual average concentrations) and NDVI (based on data from 2003) and insulin resistance. Gam models adjusted for city, cohort, sex, age, BMI.

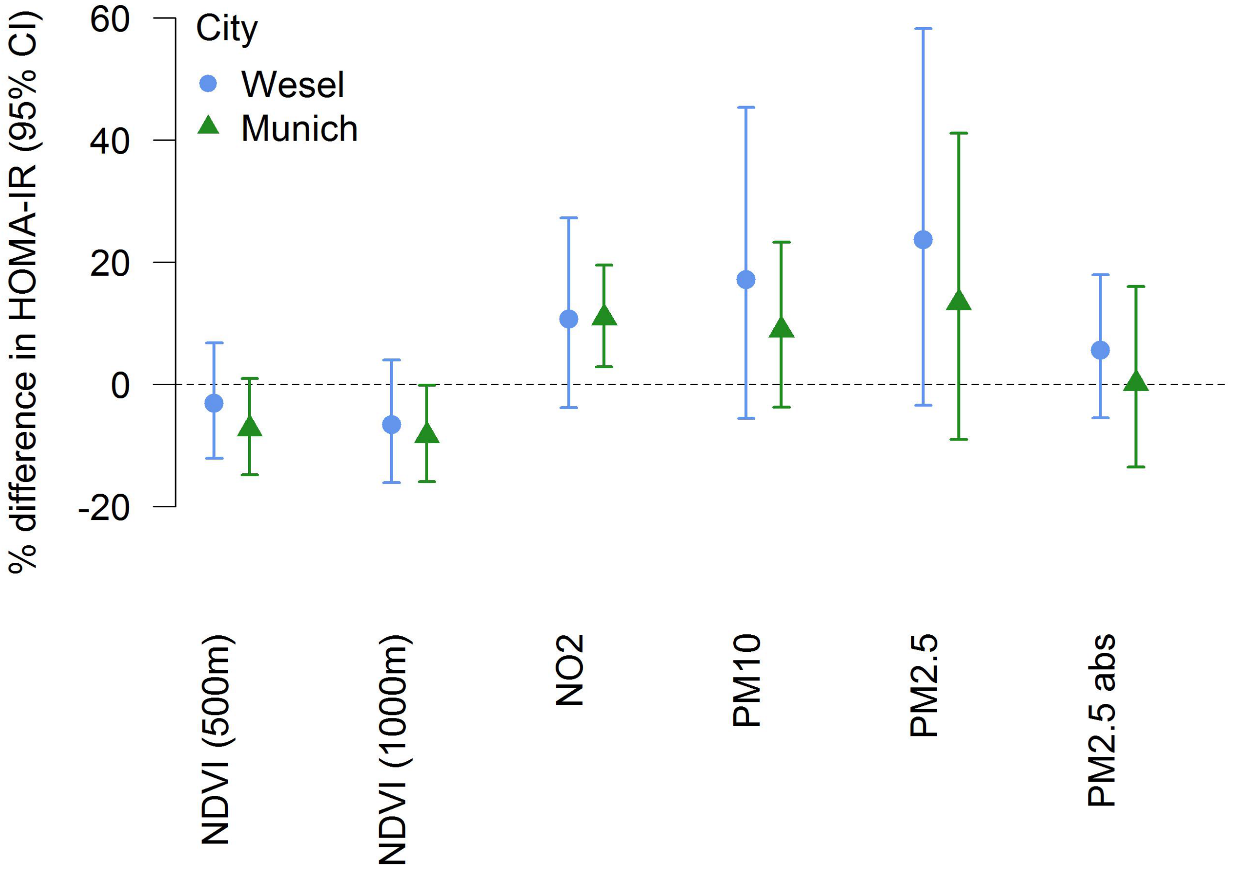

Figure S3. Comparable effect estimates in both cities. Gam models adjusted for cohort, sex, age, BMI, smoking by the adolescent, paternal and maternal education level, secondhand smoke in the home, physical activity, pubertal state, city-specific equivalent net income tertiles. P-values for the interaction with study area: NDVI (500m): p=0.241, NDVI (1000m): p=0.475, NO_2: p=0.3077, PM_{10}: p=0.655, $PM_{2.5}$=0.718, $PM_{2.5}$ abs.: p=0.816.

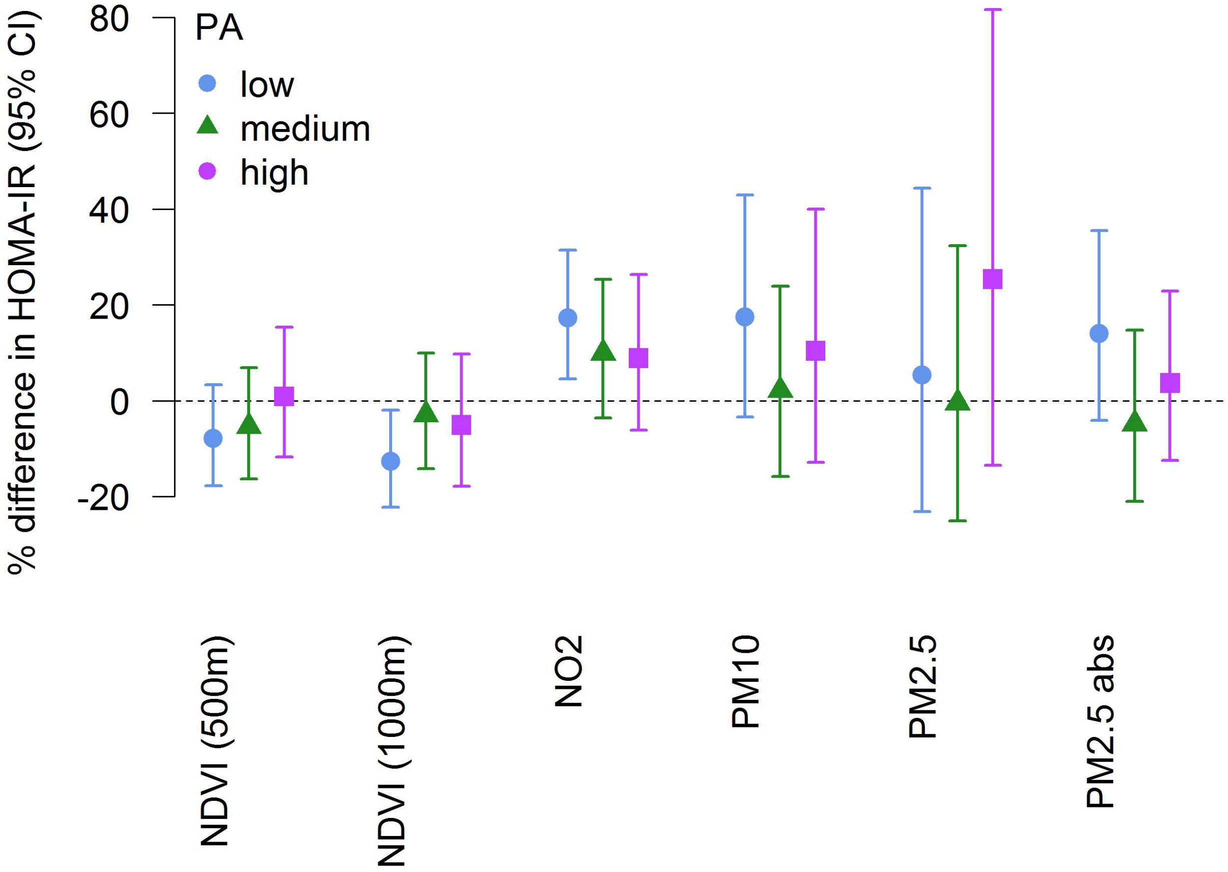

Figure S4. Gam models stratified for physical activity level adjusted for cohort, city, sex, age, BMI, smoking by the adolescent, paternal and maternal education level, secondhand smoke in the home, pubertal state, city-specific equivalent net income tertiles. P-values for the interaction with physical activity: NDVI (500m): p=0.933, NDVI (1000m): p=0.966, NO_2: p=0.378, PM_{10}: p=0.433, $PM_{2.5}$=0.474, $PM_{2.5}$ abs.: p=0.926.

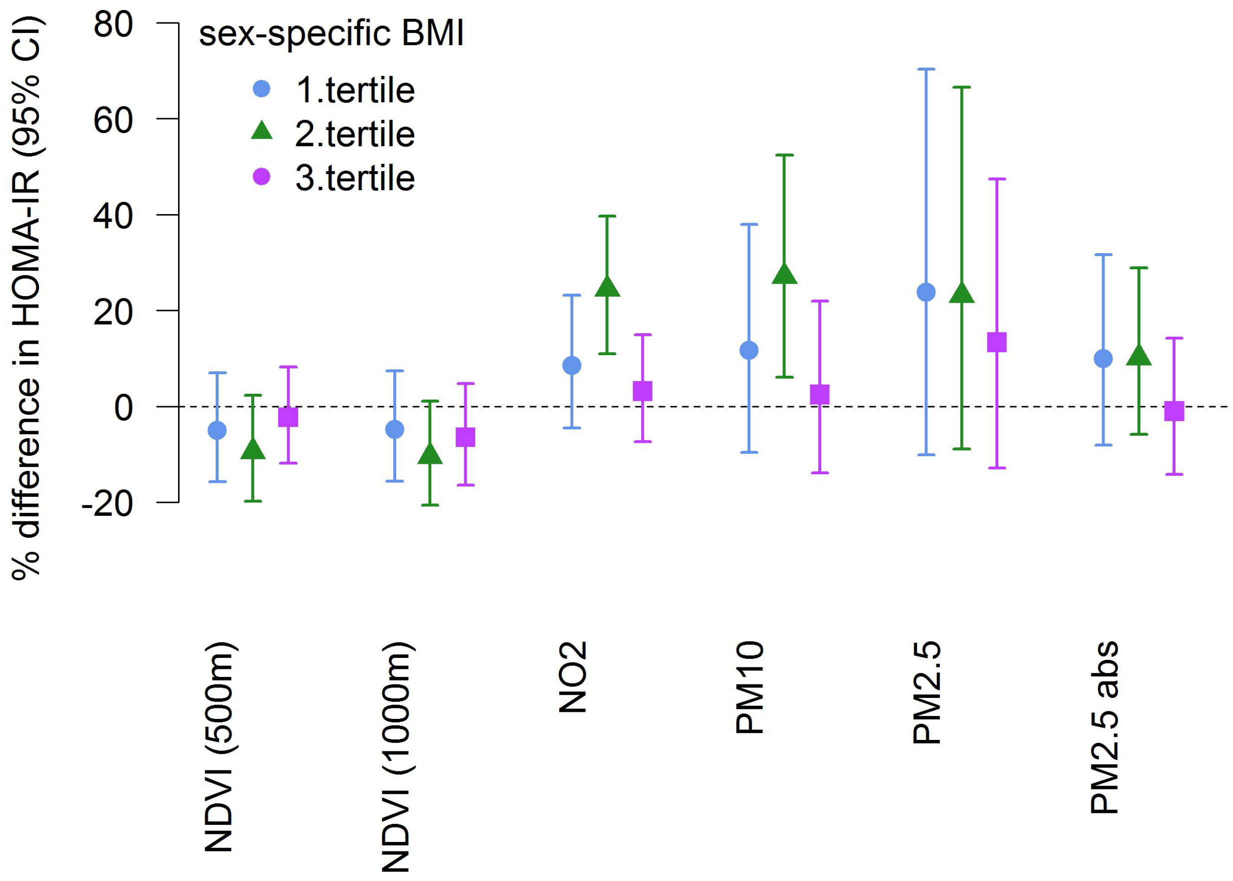

Figure S5. Gam models stratified for sex-specific BMI tertiles adjusted for cohort, city, sex, age, smoking by the adolescent, paternal and maternal education level, secondhand smoke in the home, pubertal state, physical activity, city-specific equivalent net income tertiles. P-values for the interaction with BMI: NDVI (500m): p=0.432, NDVI (1000m): p=0.668, NO_2: p=0.788, PM_{10}: p=0.630, $PM_{2.5}$=0.585, $PM_{2.5}$ abs.: p=0.107.
